# Quantitative Cherenkov‐excited fluorescence emission dosimetry for HDR Co‐60 brachytherapy: Calibration and planar validation against TPS

**DOI:** 10.1002/acm2.70785

**Published:** 2026-09-09

**Authors:** Yagiz Yedekci, Mehmet Fazıl Enkavi, Ferah Yildiz

**Affiliations:** ^1^ Department of Radiation Oncology, Faculty of Medicine Hacettepe University Ankara Turkey; ^2^ Department of Radiation Oncology Medicana International Ankara Hospitals Ankara Turkey

**Keywords:** 60Co brachytherapy, Cherenkov emission, fluorescence, optical dosimetry, quality assurance

## Abstract

**Background:**

Accurate dose verification in High‐Dose‐Rate (HDR) brachytherapy presents a significant challenge due to steep dose gradients and the absence of beam collimation. Conventional quality assurance (QA) methods typically rely on point detectors or film‐based techniques, which lack the capability to provide real‐time, full‐field visualization of dose distributions in a water‐equivalent medium. While Cherenkov emission (CE) imaging has been investigated for Iridium‐192 (^192^Ir) systems, the low mean photon energy of ^192^Ir (∼380 keV) constrains the optical yield and limits the signal‐to‐noise ratio. Because Cobalt‐60 (^60^Co) emits higher primary photon energies, it generates distinct Cherenkov intensities and spatial characteristics, requiring dedicated evaluation.

**Purpose:**

This study aims to characterize the relationship between Cherenkov emission intensity and absorbed dose generated by an HDR ^60^Co source. Furthermore, it seeks to generate high‐resolution two‐dimensional dose maps using Cherenkov‐excited fluorescence imaging and validate their dosimetric accuracy against Treatment Planning System (TPS) calculations to evaluate the system for clinical QA implementation.

**Methods:**

A custom 3D‐printed polylactic acid (PLA) phantom was fabricated with an internal cavity filled with an aqueous quinine hemisulfate solution (1 g/L). This solution was used to convert ultraviolet Cherenkov photons into isotropic visible fluorescence, thereby enhancing optical signal detection. Optical emissions were captured using a standard monochrome CMOS camera positioned at a 24 cm working distance. To eliminate radiation‐induced artifacts on the sensor without relying on complex time‐gated hardware, an image processing pipeline comprising dark frame subtraction, median filtering, and morphological operations was applied. A calibration function was established by delivering doses between 10 and 1500 cGy using a ^60^Co HDR afterloader. Dosimetric accuracy was then evaluated across ten independent verification plans using two‐dimensional gamma index analysis with 3% dose difference and 3 mm distance‐to‐agreement (3%/3 mm) criteria.

**Results:**

The dosimetric calibration demonstrated a highly predictable and linear dose‐response relationship, yielding a coefficient of determination (*R* ^2^) of 0.9999 and a root mean square error (RMSE) of 3.46 cGy. For the clinical verification plans, the CE‐derived 2D dose maps exhibited strong spatial agreement with the TPS calculations. Gamma passing rates across the ten plans ranged from 91.6% to 97.3%, resulting in a mean passing rate of 94.2% ± 1.9% and an average mean gamma value of 0.57 ± 0.05. One‐dimensional line profiles further confirmed the high spatial fidelity of the system in accurately tracking steep dose fall‐offs.

**Conclusion:**

The integration of Cherenkov‐excited fluorescence imaging with a standard CMOS sensor provides a highly linear, practical, and cost‐effective planar dose verification tool for continuous‐emission ^60^Co HDR brachytherapy. The proposed methodology mitigates the signal limitations inherent to lower‐energy isotopes and achieves strong spatial agreement with TPS calculations, supporting its reliability as an independent QA modality in a controlled phantom environment.

## 1 INTRODUCTION

Cherenkov emission (CE) arises when a charged particle propagates through a dielectric medium with a velocity exceeding the phase velocity of light in that medium. The emission condition is defined by βn > 1, where β = v/c represents the normalized particle velocity, v is the particle speed, c is the speed of light in vacuum, and n denotes the refractive index of the medium. Under this condition, coherent electromagnetic radiation is generated along a characteristic emission angle relative to the particle trajectory. According to the Frank–Tamm formalism, the spectral distribution of CE is continuous and proportional to 1/λ^2^, where λ represents the wavelength of the emitted electromagnetic radiation. This relationship results in enhanced emission at shorter wavelengths (predominantly in the near‐ultraviolet and blue‐visible regions, typically spanning 200–600 nm), producing the characteristic blue glow observed in optically transparent materials.^1^

Although originally investigated within the context of high‐energy particle physics, CE has recently emerged as a promising optical probe in radiotherapy physics. Because the generation of Cherenkov photons is fundamentally linked to the fluence and energy distribution of secondary charged particles produced during photon or electron irradiation, the detected optical signal can serve as a surrogate for local absorbed dose. This physical coupling between radiation transport and optical emission has motivated increasing interest in CE‐based techniques for non‐invasive dose visualization and treatment verification.

In external beam radiotherapy (EBRT), CE imaging has been successfully applied for surface dose estimation, geometric verification, and real‐time treatment monitoring.^2^, ^3^, ^4^, ^5^, ^6^, ^7^, ^8^ These applications leverage the proportional relationship between optical emission intensity and deposited dose. However, the translation of CE imaging into brachytherapy remains comparatively limited, despite the unique potential advantages it offers in this context.

High‐dose‐rate (HDR) brachytherapy delivers highly localized radiation through intracavitary or interstitial placement of radioactive sources. Due to the steep dose gradients surrounding the source and the absence of beam collimation, accurate dose verification presents a significant challenge. Conventional quality assurance (QA) methods rely primarily on point detectors, source position verification systems, or film‐based techniques. While effective for specific tasks, these approaches lack the capability to provide real‐time, full‐field visualization of dose distribution in a water‐equivalent medium. An optical imaging modality capable of planar dose mapping could therefore offer a complementary and potentially transformative QA strategy for HDR brachytherapy.

Previous investigations have demonstrated the feasibility of CE imaging for Iridium‐192 (^192^Ir) HDR systems, primarily focusing on source position verification and qualitative dose visualization.^9^, ^10^ In terms of beam quality, ^192^Ir emits a complex gamma spectrum with a relatively low mean energy of approximately 380 keV. This energy is only marginally above the Cherenkov threshold in water, which is approximately 264 keV for secondary electrons.^11^ Consequently, the secondary electrons generated by ^192^Ir have velocities close to the threshold, resulting in a relatively constrained CE yield per unit absorbed dose and leading to signal‐to‐noise limitations, particularly in high‐gradient regions near the source.^9^, ^10^

In contrast, Cobalt‐60 (^60^Co) possesses a significantly higher‐energy spectrum, emitting principal primary gamma photons at 1.17 MeV and 1.33 MeV. Because the Cherenkov photon yield per unit path length follows the Frank–Tamm relation and scales with $1-\frac{1}{\beta^{2}n^{2}}$, CE production is intrinsically dependent on the energy distribution of secondary electrons rather than solely on absorbed dose. Consequently, differences in primary photon energy spectra between ^192^Ir and ^60^Co are expected to produce distinct CE intensities and spatial characteristics, even under equivalent dose conditions.

Therefore, findings derived from ^192^Ir‐based systems cannot be directly extrapolated to ^60^Co HDR brachytherapy. The present study provides a dedicated investigation of CE generated by an HDR ^60^Co source. Specifically, we aim to characterize the relationship between CE intensity and absorbed dose, generate high‐resolution two‐dimensional dose maps based on CE imaging, and perform gamma index analysis comparing CE‐derived measurements with Treatment Planning System (TPS) calculations to evaluate dosimetric and geometric accuracy for clinical implementation.

## 2 MATERIALS AND METHODS

### 2.1 Phantom design and experimental setup

A custom phantom was fabricated from polylactic acid (PLA) using fused deposition modeling (FDM) 3D printing with 90% infill to ensure structural stability and watertightness. The phantom was designed to incorporate an internal cavity with dimensions of 7.5 × 7.5 cm^2^ and a depth of 1 cm. This cavity was filled with an aqueous solution containing 1 g/L quinine hemisulfate monohydrate (Sigma‐Aldrich, St. Louis, MO, USA) dissolved in distilled water. Quinine was employed to enhance optical signal detection through Cherenkov‐excited fluorescence, whereby anisotropically emitted ultraviolet Cherenkov photons (typically in the 250–380 nm range) are absorbed and re‐emitted isotropically as fluorescence in the visible spectrum (peaking in the blue region at approximately 450 nm), improving detection efficiency.^12^ Although this re‐emission is isotropic, any potential optical blurring is physically minimized by the shallow 1 cm depth of the phantom cavity, which restricts lateral light diffusion. Furthermore, because the fluorescence decay of quinine occurs on a nanosecond timescale, this conversion process introduces no temporal lag, thereby preserving both the spatial fidelity and the real‐time imaging capability of the system.

**FIGURE 1:**
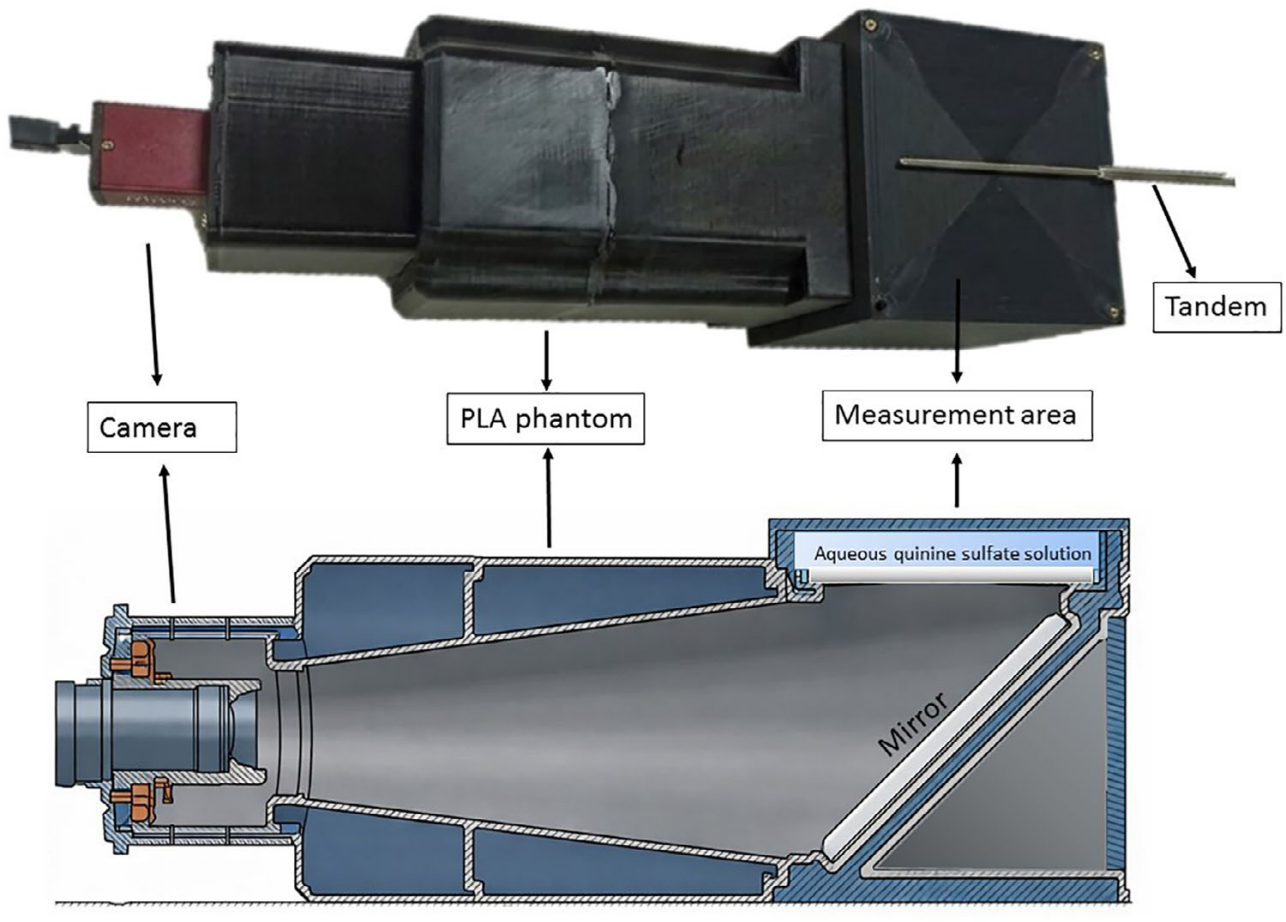
Photograph of the phantom and its corresponding cross‐sectional schematic illustration, demonstrating the overall geometry and internal cavity configuration.

For optical signal collection, a highly reflective front‐surface aluminum mirror was positioned beneath the measurement volume at an angle of 45°, redirecting emitted light toward the imaging system. The camera–lens assembly was positioned at a working distance of 24 cm from the center of the mirror. The entire setup was enclosed within a light‐tight PLA enclosure to eliminate ambient light contamination and ensure dark acquisition conditions. Figure 1 shows the photograph and the corresponding cross‐sectional schematic of the experimental phantom.

### 2.2 Optical imaging system

Cherenkov‐excited fluorescence emissions were recorded using a monochrome CMOS camera (Mako G‐234B, Allied Vision Technologies GmbH, Stadtroda, Germany) equipped with a Sony IMX249 sensor featuring a pixel size of 5.86 × 5.86 µm. A 25 mm fixed focal length lens (Edmund Optics, Barrington, NJ, USA) was mounted on the camera.

The optical geometry was configured to project a 6.8 × 6.8 cm^2^ field of view (FOV) onto a 1050 × 1050 pixel region of the sensor. This configuration provided an effective spatial resolution of 0.065 mm per pixel at the specified working distance. The lens aperture was set to f/1.4 to maximize photon collection efficiency under low‐light conditions.

Optical distortion associated with the lens–sensor combination was quantified and found to be less than 0.03 mm at the periphery of the measurement field, corresponding to less than one‐half of a pixel. This level of distortion was considered negligible relative to the system's spatial resolution and therefore did not require correction.

### 2.3 Image acquisition and processing

Image acquisition was conducted using an integration time (exposure) of 200 ms at a frame rate of 5 frames per second (fps). The sensor gain was set to 40 dB to enhance signal sensitivity under low‐light CE conditions.

Post‐processing of the acquired images was performed using the OpenCV library (Open Source Computer Vision Library). The image processing pipeline consisted of the following sequential steps:

1. Dark Frame Subtraction: A background (dark) frame acquired under identical acquisition settings without irradiation was subtracted from each raw frame to eliminate fixed pattern noise and dark current contributions.
2. Noise Reduction: A 5 × 5 median filter was applied to suppress radiation‐induced transient noise, primarily salt‐and‐pepper artifacts caused by high‐energy photon or secondary particle interactions with the CMOS sensor. This kernel size was selected based on preliminary testing of different dimensions (3 × 3, 5 × 5, and 7 × 7); a 5 × 5 window (corresponding to a physical area of 0.325 by 0.325 mm) was found to be the optimal size to fully eliminate multi‐pixel noise clusters without compromising the steep dose gradients near the source.
3. Dead Pixel Correction: A morphological opening operation using a 5 × 5 structuring element was implemented to reduce artifacts associated with dead or hot pixels while preserving spatial structures relevant for dosimetric evaluation.
4. Image Integration: The processed frames were cumulatively summed to generate a high signal‐to‐noise ratio (SNR) integrated image, which was subsequently used for quantitative dosimetric analysis.

### 2.4 Dosimetric calibration of the Cherenkov imaging system

To establish a quantitative relationship between the measured Cherenkov‐excited fluorescence intensity and the absorbed radiation dose, a systematic dosimetric calibration procedure was performed.

A tandem applicator was securely positioned in contact with the phantom surface to reproduce the clinical irradiation geometry. The phantom–applicator assembly underwent volumetric CT imaging using a CT scanner (Aquilion LB; Toshiba Medical Systems, Otawara, Japan) with a slice thickness of 5 mm. The acquired DICOM dataset was imported into the Sagiplan® TPS (Eckert & Ziegler BEBIG GmbH, Berlin, Germany) for dose calculation utilizing the standard AAPM TG‐43 formalism.

Within the TPS environment, three reference points were created along the geometric center of the optically active measurement volume. The mean dose calculated within those points was defined as the reference dosimetric quantity for calibration purposes. Calibration treatment plans were generated to produce approximately uniform dose distributions encompassing the reference region, thereby minimizing dose gradients within the analyzed optical region of interest (ROI).

**FIGURE 2:**
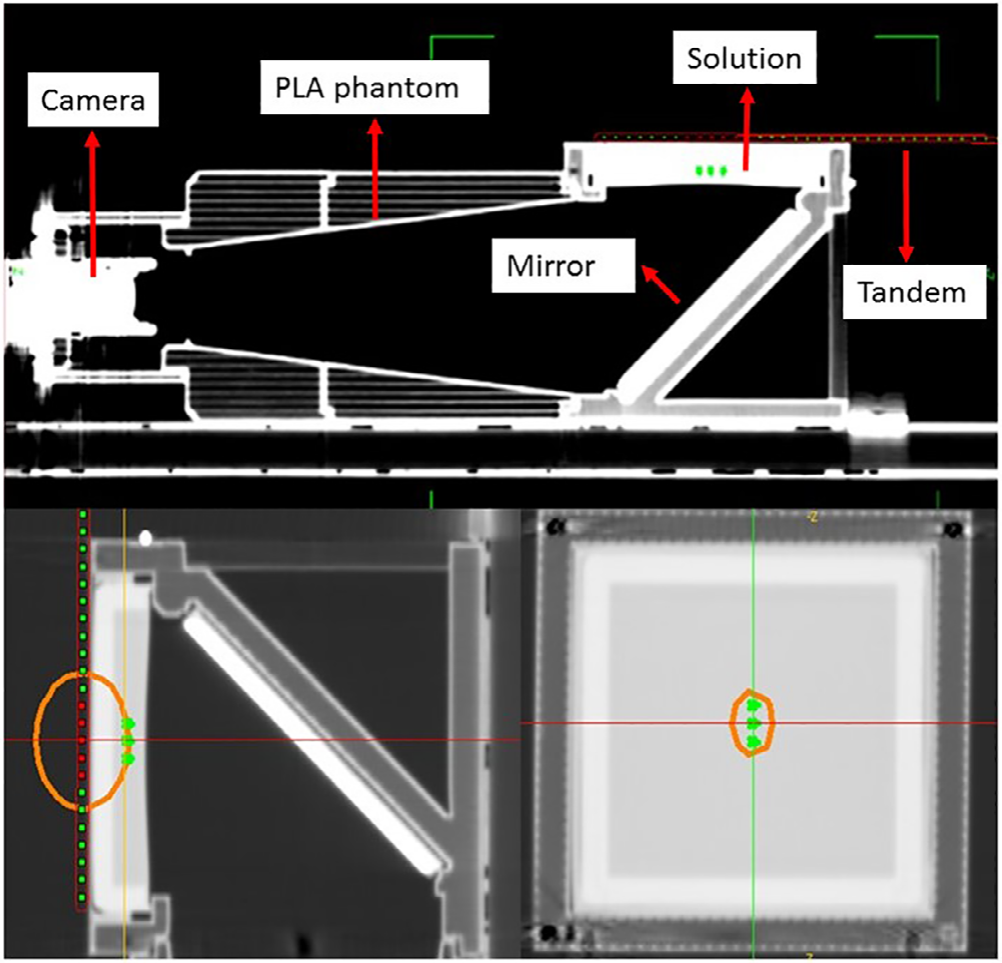
Example of the treatment plan used for calibration, illustrating the three reference points defined within the 500 cGy plan and the prescribed dose of 500 cGy delivered to each point.

Irradiations were delivered using a Saginova® High Dose Rate (HDR) afterloader (Eckert & Ziegler BEBIG GmbH, Berlin, Germany) equipped with a ^60^Co radioactive source. Ten independent calibration plans were created with prescribed doses of 10, 20, 50, 100, 200, 300, 500, 700, 1000, and 1500 cGy at the reference structure. For all ten calibration irradiations, the source active dwell positions were kept identical at positions 40, 45, 50, 55, and 60 mm. In the baseline 10 cGy plan, the corresponding dwell times for these positions were 1.0, 0.9, 0.9, 0.9, and 1.0 s, respectively. For the remaining calibration dose levels, these dwell times were scaled proportionally (e.g., 2.0, 1.8, 1.8, 1.8, and 2.0 s for the 20 cGy plan) to maintain an identical spatial dose distribution while scaling the absolute dose. These calibration plans were designed solely to establish the intensity‐to‐dose conversion and are entirely separate from the clinical verification plans used later for methodology assessment. Figure 2 shows the reference points along with the 500 cGy isodose line used in the calibration of the 500 cGy plan.

For each irradiation, optical image acquisition and processing were performed using identical parameters as described previously. A fixed two‐dimensional ROI, geometrically corresponding to the projected reference structure, was applied to the integrated optical image. The mean pixel intensity within this ROI was extracted after background correction.

The resulting intensity–dose data pairs were fitted using linear regression analysis to evaluate the proportionality between Cherenkov‐excited fluorescence intensity and absorbed dose. The goodness of fit (*R* ^2^) and residual distribution were assessed to verify linear system response within the investigated dose range. The obtained calibration function was subsequently used to convert measured optical intensity values (arbitrary units) into absolute dose values (cGy) for all experimental measurements.

### 2.5 Quantitative evaluation via gamma analysis

The dosimetric accuracy of the Cherenkov‐based optical imaging system was quantitatively evaluated using gamma index analysis.

Ten independent verification irradiation plans were generated in the TPS under clinically relevant HDR ^60^Co delivery conditions. For each plan, the planar dose distribution corresponding to the effective optical measurement depth (0.5 cm water‐equivalent depth within the measurement phantom) was exported from the TPS with a calculation grid resolution of 1 mm. These calculated dose maps served as the reference distributions.

The Cherenkov‐derived dose maps were obtained by converting calibrated optical intensity images into absolute dose using the previously established calibration function. Prior to gamma evaluation, both measured and TPS dose maps were spatially registered and resampled to a common pixel resolution using bilinear interpolation to ensure geometric consistency.

Gamma analysis was performed using a 3%/3 mm acceptance criterion. Both local and global normalization approaches were evaluated; unless otherwise stated, results are reported using global dose normalization relative to the maximum dose within the analyzed field. A dose threshold of 20% of the maximum dose was applied to exclude low‐dose regions dominated by statistical noise.

The gamma passing rate was defined as the percentage of evaluated pixels with *γ* ≤ 1. The mean gamma value and spatial gamma distribution maps were additionally analyzed to assess systematic spatial deviations between measured and calculated dose distributions.

## 3 RESULTS

**FIGURE 3:**
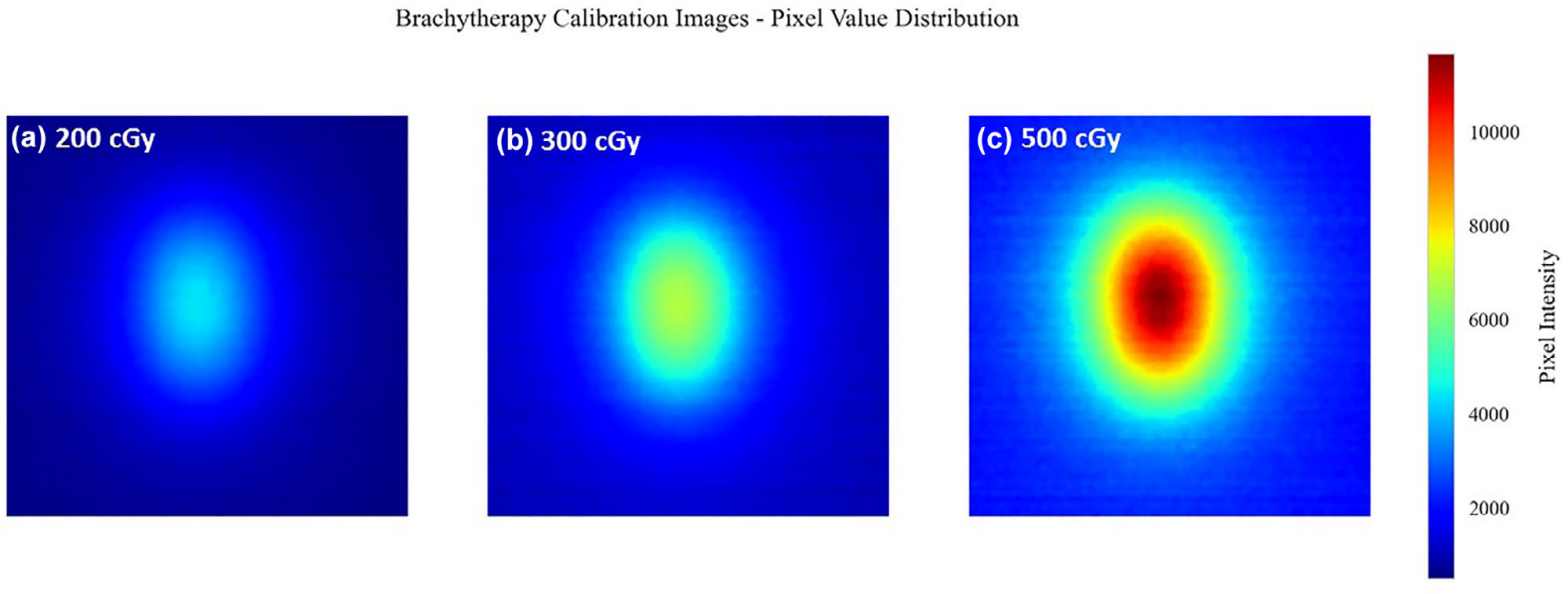
Representative Cherenkov‐excited fluorescence images obtained at different reference calibration doses: (a) 200 cGy, (b) 300 cGy, and (c) 500 cGy. The images are color‐coded to visualize the pixel intensity distribution.

Representative CE images acquired at delivered doses of 200, 300, and 500 cGy are shown in Figure 3. Pixel intensity distributions were visualized using a calibrated color scale, enabling qualitative assessment of the dose‐dependent increase in optical signal. A progressive enhancement in CE intensity was observed with increasing irradiation dose.

**FIGURE 4:**
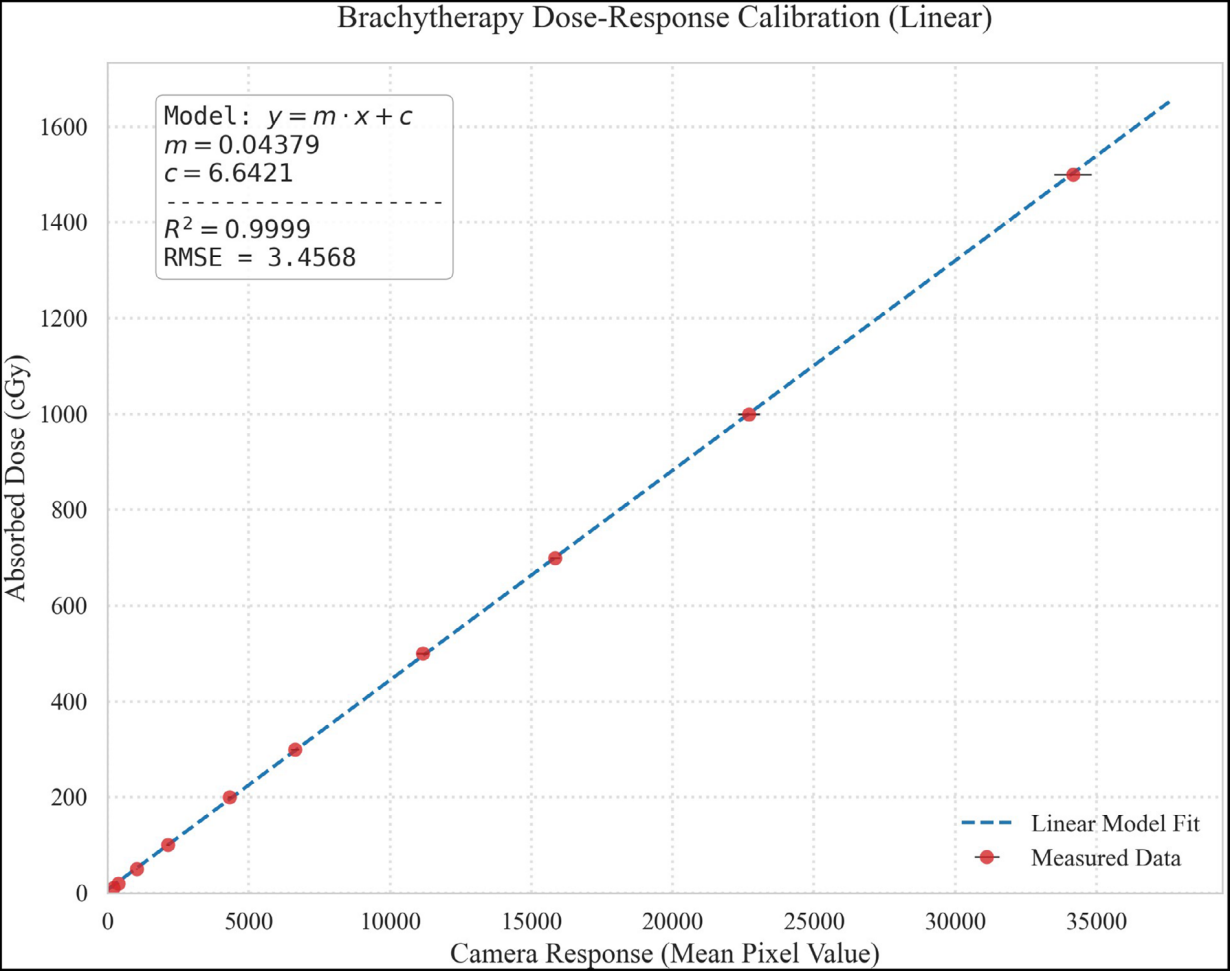
Brachytherapy dose‐response calibration curve. The red markers represent the measured mean pixel values at various absorbed dose levels (cGy), while the dashed blue line indicates the linear model fit ($R^{2}$ = 0.9999). The regression equation and corresponding error metrics are displayed within the plot.

For quantitative dose–response analysis, mean pixel intensities were extracted from centrally positioned 50 × 50 pixel regions of interest (ROIs) in each image. These values were plotted against the corresponding absorbed doses to derive a calibration function (Figure 4).

Linear regression analysis demonstrated a strong linear relationship between mean pixel intensity and delivered dose over the investigated range. Fitting the data to a first‐order model (y = mx + c) yielded a slope (m) of 0.04379 and an intercept (c) of 6.6421. The model provided an excellent description of the relationship between the TPS‐calculated reference dose and the measured mean Cherenkov‐excited fluorescence intensity, yielding a coefficient of determination (*R* ^2^) of 0.9999.

The predictive performance of the calibration model was further evaluated using the root mean square error (RMSE), which was calculated as 3.4568 cGy. These findings indicate that the camera‐derived Cherenkov‐excited fluorescence signal exhibits a highly linear and quantitatively reliable response to absorbed dose within the examined dose interval.

Following validation of the linear calibration model (*R* ^2^ = 0.9999), the methodology was further assessed using a separate set of ten independent clinical verification treatment plans delivered to the phantom. Cherenkov‐excited fluorescence images acquired during irradiation were converted into absolute two‐dimensional dose distributions using the previously derived calibration coefficients (*m* = 0.04379, *c* = 6.6421).

The resulting camera‐based dose maps were spatially registered and directly compared with the corresponding reference dose distributions exported from the TPS. This approach enabled a quantitative evaluation of the agreement between optically derived dose measurements and TPS‐calculated dose distributions under clinically relevant delivery conditions.

Dosimetric concordance was assessed using two‐dimensional gamma index analysis. Acceptance criteria were defined as a 3% dose difference (DD) and 3 mm distance‐to‐agreement (DTA). The gamma analysis results for all ten treatment plans are summarized in Table 1.

**TABLE 1:** Summary of gamma index analysis results for ten independent verification plans using 3%/3 mm acceptance criteria.

Plan #	Gamma Pass Rate (%)	Mean Gamma
1	95.2	0.56
2	94.3	0.54
3	94.1	0.61
4	96.6	0.54
5	92.6	0.55
6	91.9	0.63
7	95.7	0.56
8	91.6	0.65
9	97.3	0.49
10	92.8	0.55
Mean ± SD	94.2 ± 1.9	0.57 ± 0.05

Across the evaluated plans, the system demonstrated consistently high agreement with TPS calculations. Gamma pass rates ranged from 91.6 % to 97.3%, with a mean value of 94.2 % ± 1.9.

**FIGURE 5:**
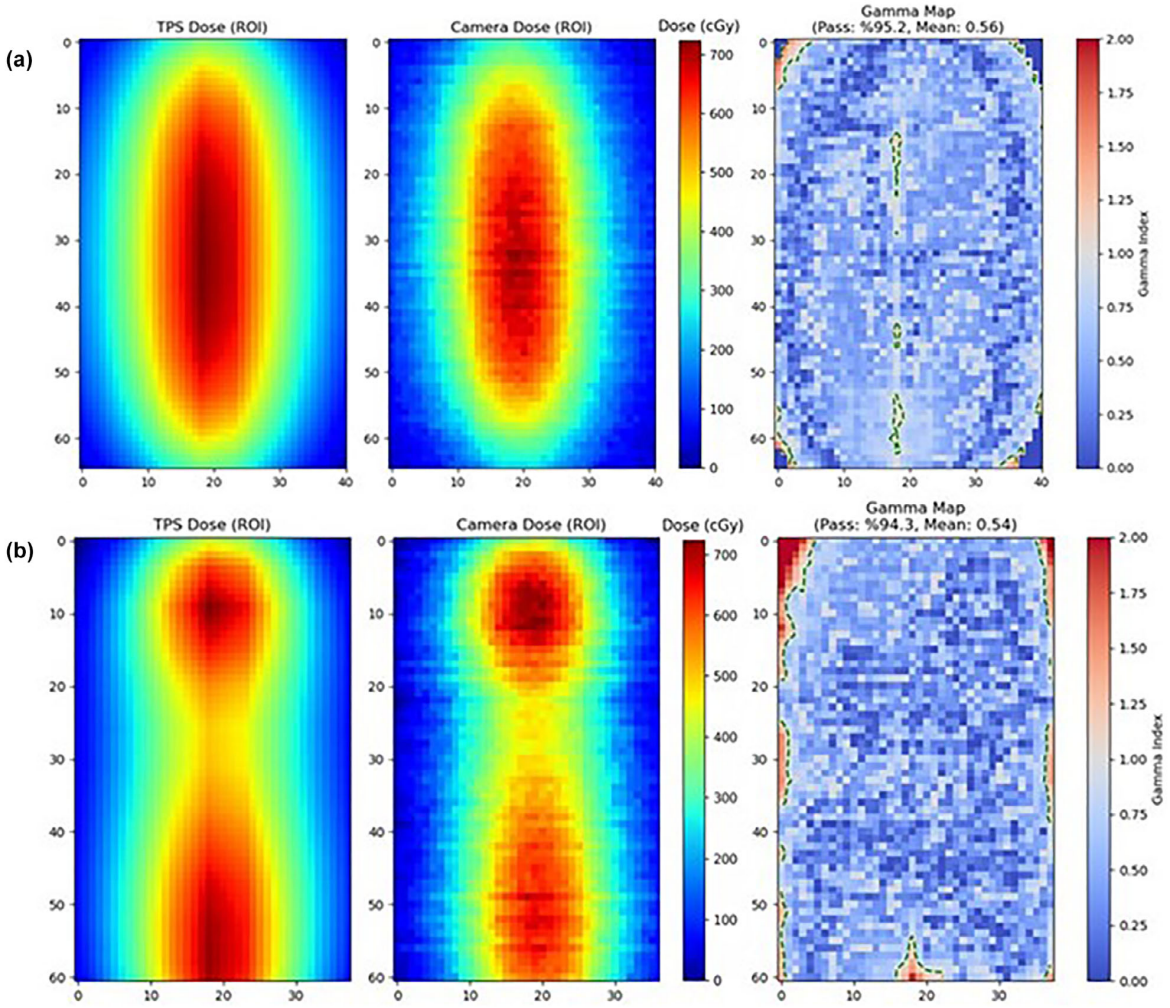
Representative spatial dose distributions and corresponding gamma analysis maps for two treatment plans. The gamma evaluation was performed using 3% dose difference (DD) and 3 mm distance‐to‐agreement (DTA) criteria. The maps predominantly exhibit index values ≤ 1 (displayed in blue/white), indicating that the majority of evaluated points satisfied the acceptance criteria.

Representative spatial dose distributions and corresponding gamma maps for two treatment plans are presented in Figure 5.

Gamma maps predominantly exhibited index values ≤ 1 (displayed in blue/white, average mean gamma:0.57 ± 0.05), indicating that the majority of evaluated points satisfied the predefined acceptance criteria. Regions exceeding the gamma threshold (index > 1) were limited in extent and were primarily located in high‐gradient regions or near phantom boundaries, where minor spatial misalignments or resolution differences are expected to have a proportionally greater impact.

**FIGURE 6:**
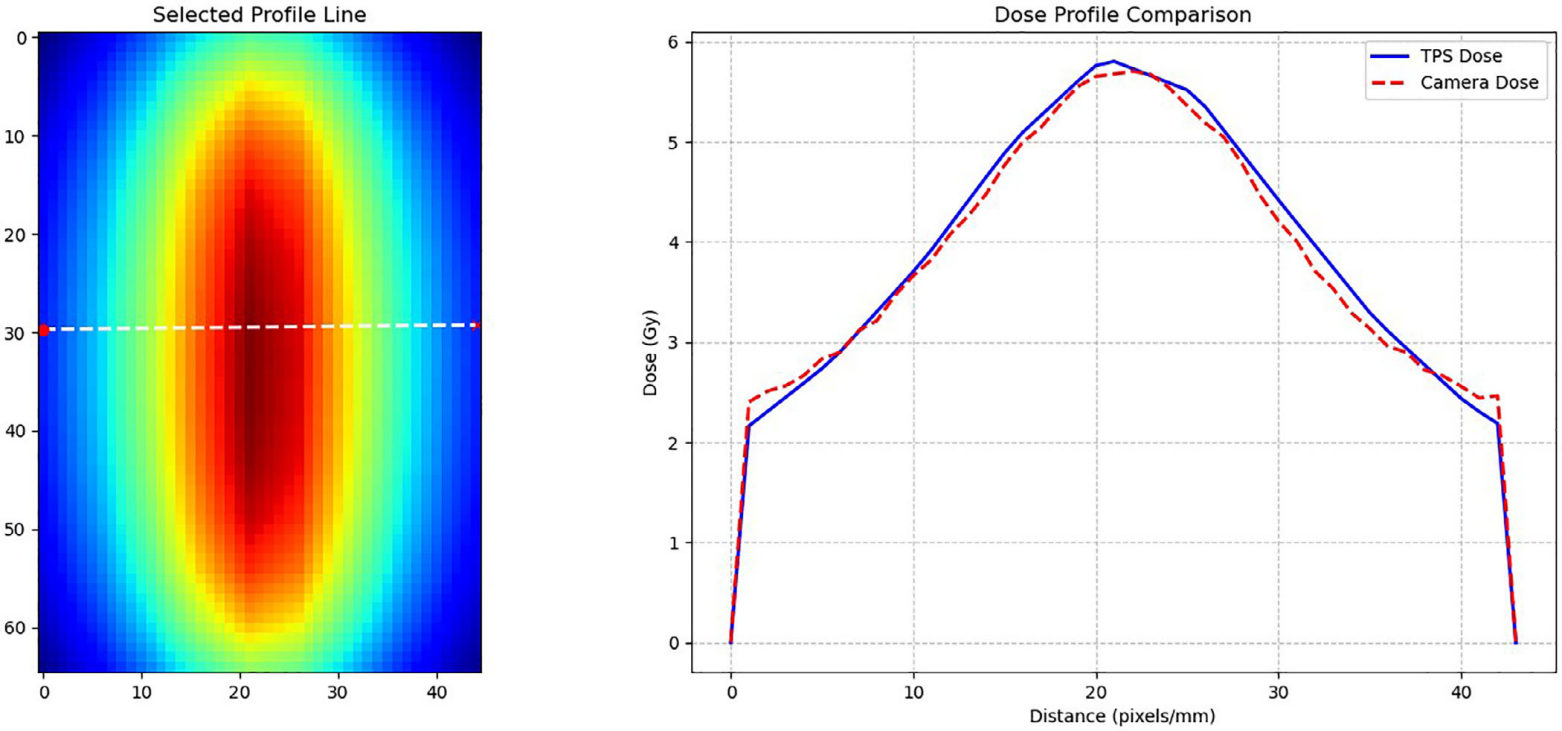
Comparison of one‐dimensional dose profiles between the treatment planning system (TPS) calculation and the Cherenkov‐based measurement for Plan 1.

To further assess the spatial accuracy and the system's performance in resolving dose gradients, a one‐dimensional line profile analysis was conducted. Figure 6 illustrates the comparison between the optically measured dose profile and the TPS‐calculated reference for Plan 1. The measured data demonstrates strong concordance with the TPS, accurately tracking the dose fall‐off and confirming the spatial fidelity of the Cherenkov imaging system, consistent with the high gamma passing rates observed in the planar analysis.

## 4 DISCUSSION

In the present study, we demonstrated the feasibility of using CE imaging for dosimetric evaluation by establishing a quantitative calibration model. Our calibration results revealed a highly linear, dose‐dependent response (*R* ^2^ = 0.9999), indicating a strong proportional relationship between the captured optical signal and the absorbed dose. This robust linearity aligns well with the theoretical formalism for CE‐to‐dose conversion established by Zlateva et al.^13^ While their Monte Carlo and experimental investigations highlighted that the CE‐to‐dose conversion factor (k_c_) for primary electron beams is highly sensitive to depth, beam quality, and measurement angle due to the continuous scattering of electrons, photon beams exhibit fundamentally different behavior. As noted in their study, photon beams—such as the high‐energy emissions from our ^60^Co source—benefit from transient charged particle equilibrium in water. This equilibrium maintains a relatively stable secondary electron fluence spectrum and CE angular distribution across varying depths. Consequently, the excellent dosimetric concordance and minimal spatial variance observed in our ^60^Co planar measurements empirically validate that the optical CE signal can be reliably and directly converted to absorbed dose without the severe depth‐ and angle‐dependent perturbations that complicate electron beam dosimetry.

Another critical challenge in Cherenkov‐based dosimetry is the degradation of image quality due to stray high‐energy radiation interacting directly with the optical sensor. As highlighted by Bruza et al.,^14^ imaging in clinical environments typically requires advanced and complex hardware, such as time‐gated Single‐Photon Avalanche Diode (SPAD) arrays, to effectively suppress ambient light and reject radiation‐induced impulse noise. While such sophisticated Geiger‐mode sensors offer excellent noise rejection for EBRT, our study demonstrates that highly accurate Cherenkov dose mapping in HDR ^60^Co brachytherapy can be achieved using a standard, cost‐effective CMOS sensor combined with a robust computational pipeline. By utilizing a light‐tight phantom enclosure to eliminate ambient light and applying dark frame subtraction alongside a median filter, we effectively mitigated the salt‐and‐pepper artifacts caused by secondary particle interactions on the sensor. The high gamma pass rates (mean 94.2%) and low RMSE (3.45 cGy) obtained in our quantitative analysis indicate that this image processing strategy successfully preserves dosimetric fidelity. Consequently, our methodology provides a practical and highly accessible alternative for routine QA applications, eliminating the immediate need for complex temporal gating hardware in a controlled phantom setting.

The application of Cherenkov imaging for HDR brachytherapy QA was previously demonstrated by Yogo et al.,^9^ who established the feasibility of optical dose mapping using an ^192^Ir source. However, as they noted, the mean photon energy of ^192^Ir (∼380 keV) is only marginally above the CE threshold in water (264 keV), resulting in a relatively constrained optical signal. Furthermore, their pure‐water setup exhibited dose discrepancies exceeding 10% in regions proximal to the source (< 7 mm). Our study builds upon this foundation by evaluating a ^60^Co source, whose significantly higher principal gamma energies (1.17 and 1.33 MeV) generate a much larger fraction of secondary electrons that comfortably exceed the Cherenkov threshold. This fundamental physical difference intrinsically enhances the Cherenkov photon yield. By coupling this robust primary emission with a quinine‐doped phantom to further amplify the signal through Cherenkov‐excited fluorescence, our system effectively mitigated the signal‐to‐noise limitations inherent to lower‐energy isotopes. Consequently, our methodology achieved excellent spatial agreement with the TPS, as evidenced by the high 3%/3 mm gamma passing rates (mean 94.2%), underscoring the distinct dosimetric reliability of optical imaging specifically tailored for ^60^Co HDR brachytherapy systems. Importantly, this fluorescence conversion process did not introduce any detectable degradation in either the spatial or temporal resolution of the dosimetry system. Although the fluorescence emission is isotropic, any potential optical blurring was physically constrained by the shallow 1 cm depth of the phantom cavity, thereby preserving the high effective spatial resolution of 0.065 mm per pixel. This high spatial fidelity is directly supported by our one‐dimensional profiles and high gamma passing rates in steep gradient regions. Temporally, because the fluorescence decay of quinine occurs on a nanosecond timescale, the conversion introduced no optical lag, fully maintaining the integrity of the 5 FPS real‐time movie mode acquisition.

The clinical translation of Cherenkov imaging for routine QA continues to evolve rapidly, as evidenced by recent advancements in EBRT. A study by Okamoto et al. ^15^ demonstrated the utility of CE for simultaneously verifying geometric parameters, such as isocenter positioning and gantry angles, in linear accelerators. However, their findings also highlighted a persistent challenge in the field: the quantification of absolute dose from Cherenkov counts exhibited notable variability (2.7% standard deviation), leading them to conclude that the reliability of Cherenkov signals for absolute dosimetry remains an open issue. Our study directly addresses this ongoing challenge within the context of HDR brachytherapy. A fundamental physical distinction driving our system's stability is the continuous nature of ^60^Co radioactive decay, which contrasts sharply with the pulsed radiation output of linear accelerators. In LINAC‐based setups, pulse‐to‐pulse dose variations and the complex temporal synchronization required for time‐gated imaging inherently introduce signal fluctuations. Conversely, the continuous and predictable photon emission from our ^60^Co source allows for steady optical signal integration over the camera's exposure time. By leveraging this inherently stable emission, alongside the amplified optical signal from our quinine‐doped phantom and a robust background subtraction pipeline, we achieved an exceptionally predictable dose‐response calibration (*R* ^2^ = 0.9999). This high dosimetric fidelity, confirmed by our 94.2% mean gamma passing rate, suggests that while absolute Cherenkov dosimetry may face limitations in dynamic, pulsed external beam environments, it is highly accurate, reproducible, and clinically viable for continuous‐emission ^60^Co HDR brachytherapy QA.

To contextualize this technology within the standard HDR clinical workflow, the developed system is envisioned as a rapid, practical, and comprehensive QA workstation that bridges the gap between conventional point detectors and film dosimetry. In a routine clinical setting, this system can be seamlessly integrated into several checkpoints: *Patient‐Specific Plan QA*, where complex treatment plans are verified against TPS calculations within minutes; *Daily Machine QA*, to rapidly verify output consistency and timer linearity using the cost‐effective CMOS sensor setup; *and Source Exchange QA*. During source exchanges, the system's exceptionally linear absolute dose‐response can provide an independent check of the new source's activity, while its high spatial and temporal resolution simultaneously verifies the mechanical positioning and step‐size accuracy of the newly installed source. This multi‐purpose versatility minimizes clinical workload while introducing a superior layer of safety through direct verification in a water‐equivalent medium.

Furthermore, while the current experimental validation was demonstrated using a single tandem applicator geometry, the inherent flexibility of the platform allows it to be readily adapted to other common gynecological brachytherapy applicators, such as tandem and ring (T&R) or tandem and ovoids (T&O). The fused deposition modeling (FDM) 3D‐printing technique used to fabricate the phantom enables the rapid customization of the top plate or phantom lid. Customized plates can be printed with contoured, form‐fitting grooves designed to rigidly and reproducibly lock a ring or ovoid assembly into a fixed position relative to the measurement cavity. Because the optical imaging system captures a wide 6.8 × 6.8 cm^2^ field of view, it is fully capable of encompassing the lateral dose spread characteristic of ring and ovoid geometries. While a flat planar cavity is highly suited for assessing the linear dose profile of a tandem, evaluating the highly three‐dimensional dose distributions of T&R or T&O assemblies would benefit from future multi‐planar phantom cavities or conformal curved imaging interfaces filled with the quinine‐doped solution. Such expansions would enable comprehensive, multi‐channel applicator QA within a single, streamlined optical workstation.

Despite the encouraging dosimetric accuracy demonstrated in this study, several important limitations warrant careful consideration. First, the current validation was performed in a two‐dimensional planar geometry at a single effective measurement depth. HDR brachytherapy inherently produces highly heterogeneous three‐dimensional dose distributions with steep radial gradients surrounding the source. While planar mapping provides meaningful spatial verification, it does not fully capture volumetric dose behavior. Future investigations should explore multi‐depth acquisitions, tomographic reconstruction approaches, or multi‐angle optical projections to enable true three‐dimensional Cherenkov‐based dosimetry. Second, signal enhancement was achieved through the use of quinine hemisulfate, which converts ultraviolet CE into visible fluorescence. Although this approach substantially improves detectability under low‐light conditions, the long‐term radiolytic stability of quinine and the potential for photobleaching under repeated high‐dose exposures remain incompletely characterized. Systematic evaluation of fluorescence stability, concentration dependence, and cumulative dose effects will be necessary to ensure long‐term reproducibility in routine QA applications. Third, all measurements were conducted in a homogeneous, optically transparent aqueous phantom. In clinical in vivo environments, tissue heterogeneity, depth‐dependent scattering and absorption, and spatially varying refractive indices would alter photon transport and potentially distort the relationship between detected optical intensity and local absorbed dose. Therefore, the present system should be interpreted as a controlled phantom‐based QA platform rather than a direct surrogate for in vivo dosimetry without additional correction models. The observed variations in gamma passing rates among the ten verification plans, particularly for Plan 6 (91.9%) and Plan 8 (91.6%), can be primarily attributed to localized discrepancies in high‐dose‐gradient regions and near phantom boundaries. Because brachytherapy doses exhibit steep radial gradients near the active source, even sub‐millimeter spatial misalignments between the physical applicator setup and the virtual TPS geometry can lead to localized gamma failures. Furthermore, optical boundary effects, such as minor light reflections or refractions at the PLA‐liquid interface, as well as resolution resampling from the camera's high resolution (0.065 mm) to the TPS grid (1.0 mm), represent additional contributing factors to these plan‐to‐plan variations.

In addition to addressing these limitations, the temporal characteristics of the developed system offer significant opportunities for future expansion. Because the optical signal was recorded in movie mode at a high frame rate of 5 frames per second (corresponding to a 200 ms integration time per frame), the raw dataset inherently possesses high temporal resolution. While the current study focused on validating the cumulative planar dose distribution, this 5 FPS acquisition rate is fully capable of resolving individual source dwell positions, real‐time dwell times, and dwell‐specific dose contributions. By tracking the spatial centroid of the fluorescence peak frame‐by‐frame, and analyzing the temporal plateau length at each active step, the system could be developed into a dynamic QA platform capable of verifying afterloader source trajectory and individual channel dwell times in real‐time.

Collectively, these limitations do not undermine the demonstrated feasibility of Cherenkov‐based planar dose verification for HDR ^60^Co brachytherapy; rather, they delineate the boundaries within which the current system provides reliable quantitative performance and define clear directions for future technical refinement.

## 5 CONCLUSION

This study demonstrates the clinical feasibility and quantitative dosimetric performance of CE imaging as a QA tool for ^60^Co HDR brachytherapy. By exploiting the high‐energy photon emissions of ^60^Co in combination with a quinine‐doped phantom, we established a highly stable and linear dose–response calibration (*R* ^2^ = 0.9999). The optically derived two‐dimensional dose maps showed strong spatial agreement with TPS calculations, achieving a mean gamma passing rate of 94.2% under 3%/3 mm acceptance criteria.

Importantly, the use of a standard CMOS sensor integrated with a robust image‐processing pipeline provides a practical and cost‐effective alternative to complex time‐gated detection systems. Although the present implementation is limited to planar dose verification in a controlled phantom environment, the high level of dosimetric agreement observed supports the reliability of optical Cherenkov imaging as an independent QA modality. These findings lay the groundwork for future development toward multi‐depth and potentially volumetric Cherenkov‐based dosimetry in HDR brachytherapy.

## AUTHOR CONTRIBUTIONS

Yagiz Yedekci contributed to the study conception and design, data collection, analysis, and manuscript preparation. Mehmet Fazıl Enkavi and Ferah Yildiz contributed to the methodology, validation, supervision, and critical review of the manuscript. All authors read and approved the final manuscript.

## CONFLICT OF INTEREST STATEMENT

The authors declare no conflicts of interest or competing interest.

## ETHICAL APPROVAL

Ethical approval for this study was obtained from the institutional ethics committee.

## Data Availability

The data that support the findings of this study are available from the corresponding author upon reasonable request.
